# Can diabetes patients seeking a second hospital get better care? Results from nested case–control study

**DOI:** 10.1371/journal.pone.0210809

**Published:** 2019-01-22

**Authors:** Jae-Hyun Kim, Eun-Cheol Park

**Affiliations:** 1 Department of Health Administration, College of Health Science, Dankook University, Cheonan, Republic of Korea; 2 Institute of Health Promotion and Policy, Dankook University, Cheonan, Republic of Korea; 3 Institute of Health Services Research, Yonsei University, Seoul, Republic of Korea; 4 Department of Preventive Medicine, Yonsei University College of Medicine, Seoul, Republic of Korea; University of Adelaide School of Medicine, AUSTRALIA

## Abstract

This study investigates the effects of the number of medical institutions visited on risk of death. This study conducted a nested case-control design using the National Health Insurance Service–Senior database from 2002 to 2013. Cases were defined as those with death among outpatients who had first diagnosis of diabetes mellitus (E10-E14) after entry into the base cohort and controls were selected by incidence density sampling and matched to cases based on age, and sex. Our main results were presented by conditional logistic regression for nested case-controls design. Of total 55,558 final study samples, there were 9,313 (16.8%) cases and 46,245 (83.2%) controls. With an increase by one point in the number of hospitals per medical utilization, risk of death significantly increased by 4.1% (odds ratio (OR): 1.041, 95% confidence interval [CI]: 1.039–1.043). In both medical utilization and number of hospitals, those with high medical utilization (OR: 1.065, 95% CI: 1.059–1.070) and number of hospitals (OR: 1.049, 95% CI: 1.041–1.058) for risk of death were significantly higher than those with low medical utilization (OR: 1.040, 95% CI: 1.037–1.043) and number of hospitals (OR: 1.029, 95% CI: 1.027–1.032), respectively. The number of medical institution visited was significantly associated with risk of death. Therefore, diabetics should be warned about the potential of risk of death incurred from excessive access to medical utilizations.

## Introduction

Diabetes mellitus is one of the foremost public health issues worldwide, especially in Korea which affects more than 25% of people over 60 [1]. It could increase the risk of chronic diseases such as cardiovascular disease,[2] retinopathy[3], renal failure,[4] and peripheral vascular disease and become a significant cause of morbidity and mortality in Korea[5].

Patients with chronic diseases can visit medical institutions freely under the national health insurance system in Korea. As a result, there are many patients who visit various medical facilities for the same symptom. Previous Korean study[6] of people aged ≥45 years showed that number of physician visits of those with diabetes was 8.99 per year and they had 3.02 times greater odds ratio of ≥6 physician visits compared with those with neither diabetes nor depression. In Japan, similar to medical system in Korea, more than half of the patients attending university hospitals go to multiple medical facilities [7]. In Taiwan, after the National Health Insurance (NHI) program was implemented, some studies found that not having continuity of care in provider visits frequently occurred under this system, because of the lack of restrictions, low costs, and reduction of barriers to access[8].

Previous study shows that not having continuity of care is common among patients with chronic diseases [9], especially in Asian countries where primary care doctor is not well-established [10]. These behaviors can cause increase in medical expenses [11], waste in medical resources [12], and decline in quality of continuous care [13]. Moreover, these patients are more likely to receive duplicate medications and suffer adverse drug reactions [14], although the drug utilization review (DUR) system that determines whether patients receive, or are appropriately prescribed for patient safety was introduced in Dec 2010 in Korea [15]. As a result, it is more likely to deteriorate overall health of patients [16] and in terms of continuity, comprehensiveness and coordination of care, it is particularly important for people with chronic conditions, because they often require long-term continuity of care [17].

However, to our knowledge, little has been reported about the relationship between having a continuity of care provider and their effects on survival rates of diabetes patients through nested case-control study design in Korea, although it is obvious that not having a continuity of care could induce wastefulness and adverse outcome. Therefore, the current study aimed to explore the effects of visits of medical care provider on mortality using representative database from 2002 to 2013 Korean National Health insurance service-senior (NHIS-Senior).

## Methods

### Data source

We conducted a retrospective population based nested case-control study from the National Health Insurance Service–Senior (NHIS-Senior) claim database from 2002 to 2013 released by the National Health Insurance Service in South Korea. NHIS-Senior was established by stratified systematic random sampling method to generate a representative sample in 2002 and were followed up until 2013 (12 years), and a representative sample cohort of 558,147 participants was randomly selected, comprising approximately 10% of the 60 years and above eligible Korean population in 2002. All database was linked anonymously using unique encrypted patient codes, in accordance with Korean laws on privacy. Approval from an ethics committee is not required to analyze encrypted claim data.[18]

### Study population & identification of cases

For the analysis, we included diabetes mellitus outpatients based on primary diagnosis code (E10-E14) from the International Classification of Disease 10th revision (ICD-10). From the main cohort, we conducted a nested case-control analysis. Cases for the study were identified as those with event of death among outpatients who had first diagnosis of diabetes mellitus after entry into the base cohort. The event date for the cases was defined as the date of the first case event. The cases were ascertained any time between the study entry date (Jan 1, 2002) and the study end date (December 31, 2013). Of the 55,558 final study samples, there were 9,313 cases and 46,245 controls.

### Identification of controls

In order to obtain unbiased estimates of relative risk, controls were selected by incidence density sampling from the study base, which involves matching each case to a sample of those who are at risk at the time of case occurrence.[19] Controls for the study were individuals who remained in the risk set on the date of death occurrence for the corresponding case during the study period. Controls were matched to cases based on age and sex of diabetes mellitus patients. Five controls were randomly selected for each case from the pool of all eligible controls.

### Independent variable

Number of hospital per medical utilization, our independent variable of interest, was calculated by dividing the number of hospital by participant`s total medical utilization during the follow-up period.

### Control variables

The present analyses included residential region, income, charlson comorbidity index (CCI), primary diagnosis of diabetes mellitus, type of insurance and severity of disability as control factors; all of the covariates were categorical. Residential region was categorized into metropolitan (Seoul), urban (Daejeon, Daegu, Busan, Incheon, Kwangju, or Ulsan), and rural (otherwise). CCI, which is a case mix index for adjustment of comorbidities, was grouped as scores of 0, 1, 2, and over 3. Income has deciles distribution and categorized into three groups: low (≤3), middle (4–7) or High (8–10). Primary diagnosis of diabetes mellitus was categorized into five groups: insulin-dependent diabetes mellitus (E10), non-insulin-dependent diabetes mellitus (E11), malnutrition-related diabetes mellitus (E12), other specified diabetes mellitus (E13) and unspecified diabetes mellitus (E14). Type of health insurance covered by universal coverage was divided into community insurance, workplace insurance and medical aid. Medical aid which is a social safety net similar to Medicaid or Medicare in the USA and severity of disability was compensated by the government for their medical expenses, excluding co-payments, as established by law. Based on the Korean guidelines for assessment of disability, severity of disability is divided into six grades and it was classified into three groups: nothing, moderate (1–2), severe (3–6).

### Statistical analysis

In this study, analyses were conducted with SAS statistical software (version 9.4; SAS Institute, Cary, NC, USA), using conditional logistic regression methods for matched case–controls studies. We calculated odds ratios (ORs) with 95% confidence intervals (CIs) for each exposure variable. All estimated odds ratios were conditional on the matching factors. Differences in covariate distribution between cases and controls were evaluated using chi-square tests for categorical variables. All statistical tests were two-tailed, with the null hypothesis of no difference being rejected if p < 0.05.

## Results

Table 1 shows the general characteristics after incidence density sampling. Of 55,558 outpatients with diabetes mellitus, there were 9,313 cases (16.8%) with a death event. Mean age of (p = 0.705) and percent of male (p = 0.996) was 76.7 year-old in both case and control and 50.1%, respectively. Mean of number of hospitals per medical utilization was 19.2 (SD:16.4) in cases and 13.4 (SD: 9.4) in controls (Table 1). Table 2 presents the results of conditional logistic regression for death. Metropolitan, those with complexity disease, low income, those with insulin-dependent diabetes mellitus, medical aid beneficiaries and those with severe disability were associated with increased risks of death compared to urban, those with no complexity disease, high income, other specified diabetes mellitus, workplace insurance, and those with moderate disability, respectively. With an increase in one point in the number of hospitals per medical utilization, risks of death significantly (Odds ratio (OR): 1.041, 95% confidence interval [CI]: 1.039–1.043) increased by 4.1% after adjusting for all confounders. (Table 2).

**Table 1:** General characteristics of diabetes outpatients.

	Total		Death						P-value
			No			Yes			
	N	%	Mean/N	SD/ %†	%‡	Mean/N	SD/%†	%‡	
**Continuous variables**									
# of hospitals per medical use*100	55,558	100.0	13.4	9.4		19.2	16.4		< .0001
**Age**	55,558	100.0	76.7	7.0		76.7	7.1		0.705
**Categorical variables**									
**# of hospitals** * **hospital use**									< .0001
Low*Low	20,345	36.6	15,479	76.1	33.5	4,866	23.9	52.2	
Low*High	6,895	12.4	6,232	90.4	13.5	663	9.6	7.1	
High*Low	7,700	13.9	6,029	78.3	13.0	1,671	21.7	17.9	
High*High	20,618	37.1	18,505	89.8	40.0	2,113	10.3	22.7	
**Sex**									0.996
**Male**	27,831	50.1	23,166	83.2	50.1	4,665	16.8	50.1	
**Female**	27,727	49.9	23,079	83.2	49.9	4,648	16.8	49.9	
**Residential region**									0.008
**Metropolitan**	9,505	17.1	7,815	82.2	16.9	1,690	17.8	18.1	
**Urban**	13,778	24.8	11,537	83.7	24.9	2,241	16.3	24.1	
**Rural**	32,275	58.1	26,893	83.3	58.2	5,382	16.7	57.8	
**Income**									< .0001
**Low**	16,036	28.9	13,178	82.2	28.5	2,858	17.8	30.7	
**Middle**	13,994	25.2	11,619	83.0	25.1	2,375	17.0	25.5	
**High**	25,528	46.0	21,448	84.0	46.4	4,080	16.0	43.8	
**CCI**									< .0001
**0**	19,404	34.9	16,409	84.6	35.5	2,995	15.4	32.2	
**1**	11,178	20.1	9,572	85.6	20.7	1,606	14.4	17.2	
**2**	8,487	15.3	7,060	83.2	15.3	1,427	16.8	15.3	
**≥3**	16,489	29.7	13,204	80.1	28.6	3,285	19.9	35.3	
**Primary diagnosis**									< .0001
**Insulin-dependent diabetes mellitus**	2,628	4.7	2,008	76.4	4.3	620	23.6	6.7	
**Non-insulin-dependent diabetes mellitus**	43,949	79.1	36,766	83.7	79.5	7,183	16.3	77.1	
**Malnutrition-related diabetes mellitus**	15	0.0	11	73.3	0.0	4	26.7		
**Other specified diabetes mellitus**	1,209	2.2	1,013	83.8	2.2	196	16.2	2.1	
**Unspecified diabetes mellitus**	7,757	14.0	6,447	83.1	13.9	1,310	16.9	14.1	
**Type of insurance**									< .0001
**Community insurance**	16,957	30.5	13,886	81.9	30.0	3,071	18.1	33.0	
**Workplace insurance**	32,926	59.3	27,739	84.3	60.0	5,187	15.8	55.7	
**Medical Aid**	5,675	10.2	4,620	81.4	10.0	1,055	18.6	11.3	
**Severity of disability**									0.000
**Nothing**	54,761	98.6	45,611	83.3	98.6	9,150	16.7	98.2	
**Moderate**	244	0.4	179	73.4	0.4	65	26.6	0.7	
**Severe**	553	1.0	455	82.3	1.0	98	17.7	1.1	
Total	55,558	100.0	46,245	83.2	100.0	9,313	16.8	100.0	

#: number

*: multiply

^†^width %

^‡^length %

**Table 2:** Adjusted effect of all variable on death.

	Death		
	OR	95% CI	
# of hospitals per medical use*100	1.041	1.039	1.043
**Residential region**			
**Metropolitan**	1.089	1.014	1.170
**Urban**	1.000		
**Rural**	1.019	0.964	1.077
**Income**			
**Low**	1.091	1.022	1.165
**Middle**	1.057	0.997	1.120
**High**	1.000		
**CCI**			
**0**	1.000		
**1**	0.970	0.906	1.038
**2**	1.161	1.081	1.248
**≥3**	1.451	1.369	1.538
**Primary diagnosis**			
**Insulin-dependent diabetes mellitus**	1.516	1.262	1.822
**Non-insulin-dependent diabetes mellitus**	1.009	0.860	1.185
**Malnutrition-related diabetes mellitus**	2.185	0.670	7.124
**Other specified diabetes mellitus**	1.000		
**Unspecified diabetes mellitus**	1.014	0.855	1.201
**Type of insurance**			
**Community insurance**	1.150	1.091	1.212
**Workplace insurance**	1.000		
**Medical Aid**	1.268	1.156	1.392
**Severity of disability**			
**Nothing**	1.074	0.849	1.359
**Moderate**	1.000		
**Severe**	1.812	1.247	2.633

#: number

*: multiply

Given the base effect by medical utilization and number of hospitals, we divided hospital use and number of hospitals into low and high, using the SAS *Rank* function, which assigns low to the lowest value and high to the highest value based on the number of participants, respectively. With an increase of one point in group with low hospital use, risk of death was significantly increased by 4% (OR: 1.040, 95% CI: 1.037–1.043) and there was 6.5% increase in group with high hospital use. With an increase of one point in group with low number of hospitals, risk of death was significantly increased by 2.9% (OR: 1.029, 95% CI: 1.027–1.032) and there was 4.9% increase in group with high number of hospitals. (Table 3).

**Table 3:** Adjusted effect on death by # of hospitals and hospital use.

		Death		
		OR**	95% CI	
**Hospital use**	**Low**	1.040	1.037	1.043
	**High**	1.065	1.059	1.070
**# of hospitals**	**Low**	1.029	1.027	1.032
	**High**	1.049	1.041	1.058

**adjusted for residential region, income, CCI, primary diagnosis, type of insurance and severity of disability;

#: number

Table 4 shows the combined effect of number of hospitals and medical utilization for death. After adjusting for all confounders, ORs of low number of hospital and low medical utilization were 1.148 times higher (OR: 1.148, CI: 1.077–1.224) for the risk of death compared to those with high number of hospitals and low medical utilization. In addition, those with low number of hospitals and high medical utilization had the lowest risk of death (OR: 0.358, CI: 0.324–0.395) compared to those with high number of hospitals and low medical utilization. (Table 4).

**Table 4:** Adjusted combined effect on death.

	Death		
	OR**	95% CI	
# of hospitals * hospital use			
Low*Low	1.148	1.077	1.224
Low*High	0.358	0.324	0.395
High*Low	1.000		
High*High	0.379	0.353	0.408

**adjusted for residential region, income, CCI, primary diagnosis, type of insurance and severity of disability;

#: number,

*: multiply

## Discussion

Diabetes mellitus has experienced an explosive increase in prevalence during the last three decades in the Asian population[20]as well as Korean population[1]. In Korea, because the health care delivery system under the national health insurance coverage, with its high availability and accessibility of medical care services, places no restrictions on visits to medical institutions. This allows the excessive access to all institutions and doctors, resulting in lower continuity of care that was associated with lower quality of care, and increased health care utilization and costs [21]. Disease progression or recurrence from low quality of care may be potentially associated with not receiving timely and proper treatment. As a result, progression of disease representing a severely worsening condition may cause a strong tendency toward excessive access to medical institutions.

In this study, our results showed a significant positive correlation between excessive access to medical utilizations with risk of death among outpatients identified as having diabetes mellitus by multivariate analysis. Accordingly, it may be suggested that restriction on excessive access to medical utilizations may contribute to increasing life expectancy by improving continuity of care. Also, excessive access to medical utilizations in group with high medical utilization and group visiting various hospitals are significantly more likely to increase the risk of death. Finally, those visiting less number of medical institutions continuously had the lowest risk of death compared to those with more number of hospitals and low medical utilization. It is suggested that continuity of care is positively associated with patient outcomes[22] with accumulating knowledge of the patient’s history and values. In some Western countries, older adults have been shown to be much more trusting, express higher affiliation and communication with their physicians, and less likely to seek second opinions[23]. This implies excessive access to medical institutions may occur less frequently, if trust exists.

One potential reason that provoke this behavior in Korea is its high availability and accessibility of medical care services under the national health insurance system. Although reimbursement from the national health insurance is based on a fee-for-service, the fee is not high enough to be a barrier for people who want to visit more physicians [24]. In addition, outpatient clinics in Korea do not require an appointment for consultation, and patients can freely choose hospitals or clinics for their own convenience without any restriction. Another potential reason is that it may be associated with the local culture [25]. Previous studies reported that patients have more confidence in diagnosis received at higher-level hospitals [13] and distinct seeking of high-technology treatment options [10].

It is not only the wastage of medical resources but also a burden of medical service employees. Therefore, a reduction in unnecessary health care expenditure for practice is an important issue that needs to be addressed to provide better quality of care in Korea which will soon become a super-aged society[26]. Although no exact definition for excessive access to medical institutions exists, the behaviors lead to adverse outcomes and increase health care resource consumption.[27]

Therefore, to reduce the costs incurred by excessive access to medical institutions and to promote continuity of care in the hope of early detection and intervention, it is necessary to consider various strategies. Thus, health policy makers should devote greater attention to preventing patients from engaging in excessive access to healthcare resources rather than to establishing a definition of the term. In addition, Hagihara *et al*. found patient’s inability to understand a doctor’s explanation on the treatment itself [25]. Thus, visiting various medical institutions and consulting a doctor may increase patient’s welfare through the alleviation of anxiety or other mechanisms [25] from the perspective of patients. Therefore, doctors should be aware of the importance of accurate explanations and maintenance of good doctor-patient relationships to maximize its benefits and avoid legal issues throughout the course of the disease.[28]. Third, because patients tend to have greater confidence in a diagnosis of disease from higher-level hospitals, health policy makers and hospital managers should reassess the function of the medical resource network, and the promotion of integrated health care services should be considered to enhance the healthcare services currently available by strengthening cooperation with other hospitals or institution.

In the near future, studies are suggested to investigate the underlying factors of excessive access to medical utilizations and the impact on the depletion of healthcare resources for health policy makers to build a better health delivery system.

This study has several limitations worth noting, and caution must be taken when interpreting the study`s results or attempting to generalize our findings. First, when samples were selected for our study, ICD coding was employed. However, because selected samples only relied on ICD coding of principal diagnosis, it is difficult to validate individual ICD codes, because our data are anonymized database, making them susceptible to errors related to coding. Second, we did not have information on patient preferences for care or doctor-patient relationships, or the available support systems, all of which can affect the time from first diagnosis to scheduled treatment. Thus, the time from first diagnosis to treatment might have been underestimated. Third, this study included diabetes mellitus patients based on primary diagnosis code from the ICD-10 and attempted a case-mix adjustment through CCI to see if the number of medical institutions visited affected deaths. However, when considering the nature of the elderly patients over 60 years with a variety of chronic illnesses, it cannot be precisely distinguished whether it is largely due to preference or comorbidities of diabetic patients. Fourth, although our results that the 4.1% increase in mortality rate with each increase in the number of medical institutions is statistically significant, it is still unknown how much not having a continuity of care contributes.

Despite these limitations, the major strength of this study is its population-based and record-based nature, which assured that the results are less likely affected by selection or recall biases. In addition, the current analysis has a large sample size, which provides the study with high statistical power and precision. Our findings could help other countries to improve medical care function, and provide a continuous medical care program. These improvements could reduce the likelihood of excessive medical care utilization as well as future disease burden.

## Conclusion

In conclusion, our study shows that excessive access to medical utilizations was positively associated with the risk of death. Therefore, outpatients with diabetes mellitus should be warned about the potential risk of death incurred from excessive access to medical utilizations. In addition, based on previous studies[23], because, much more trusting, stronger sense of affiliation and communication with their physicians may contribute to cessation of duplicated medical care utilization through improved patient satisfaction and quality of care, health education programs are needed to modify unrealistic views and provide a better quality of care to patients.

## Supporting information

S1 Table Results of sensitivity analysis for adjusted effect on death (division by median). (DOCX)

S2 Table Results of sensitivity analysis for adjusted combined effect on death (division by median). (DOCX)

S1 File nhis_senior_yk_2013.sas7bdat. (SAS7BDAT)
