# Correction: Can diabetes patients seeking a second hospital get better care? Results from nested case–control study

**DOI:** 10.1371/journal.pone.0303550

**Published:** 2024-05-08

**Authors:** The PLOS ONE Editors

The corresponding author of this article [1] is hereby changed to Jae-Hyun Kim (Jaehyun@dankook.ac.kr).

There are errors in the Author Contributions of the second author, Eun-Cheol Park. The correct contributions are: Conceptualization, Methodology, Supervision.
