# Supplementary material for: Can diabetes patients seeking a second hospital get better care? Results from nested case–control study
Source: PLoS One. 2019 Jan 22;14(1):e0210809. doi: 10.1371/journal.pone.0210809 (PMC6342308; doi:10.1371/journal.pone.0210809)
Supplement: S1 Table — (DOCX) [file pone.0210809.s001.docx]

**S1 Table. Results of sensitivity analysis for adjusted effect on death (division by median)**

|  |  | **Death** | | |
| --- | --- | --- | --- | --- |
|  |  | **OR**** | **95% CI** | |
| **Hospital use** | **Low (≤45)** | 1.029 | 1.027 | 1.032 |
|  | **High(≥46)** | 1.049 | 1.207 | 2.473 |
| **# of hospitals** | **Low(≤6)** | 1.040 | 1.037 | 1.043 |
|  | **High(≥7)** | 1.070 | 1.063 | 1.077 |

**adjusted for residential region, income, CCI, primary diagnosis, type of insurance and severity of disability
