# Supplementary material for: Can diabetes patients seeking a second hospital get better care? Results from nested case–control study
Source: PLoS One. 2019 Jan 22;14(1):e0210809. doi: 10.1371/journal.pone.0210809 (PMC6342308; doi:10.1371/journal.pone.0210809)
Supplement: S2 Table — (DOCX) [file pone.0210809.s002.docx]

**S2 Table. Results of sensitivity analysis for adjusted combined effect on death (division by median)**

|  | **Death** | | |
| --- | --- | --- | --- |
|  | **OR**** | **95% CI** | |
| **# of hospitals * hospital use** |  |  |  |
| **Low(≤6)*Low(≤45)** | 1.148 | 1.077 | 1.224 |
| **Low(≤6)*High(≥46)** | 0.358 | 0.324 | 0.395 |
| **High(≥7)*Low(≤45)** | 1.000 |  |  |
| **High(≥7)*High(≥46)** | 0.379 | 0.353 | 0.408 |

**adjusted for residential region, income, CCI, primary diagnosis, type of insurance and severity of disability
